# Physical, psychological, sexual, and systemic abuse of children with disabilities in East Africa: Mapping the evidence

**DOI:** 10.1371/journal.pone.0184541

**Published:** 2017-09-11

**Authors:** Niall Winters, Laurenz Langer, Anne Geniets

**Affiliations:** 1 Department of Education, University of Oxford, Oxford, United, Kingdom; 2 Africa Centre for Evidence, University of Johannesburg, Johannesburg, South Africa; Universita degli Studi di Perugia, ITALY

## Abstract

Children with disabilities (CWDs) are at a higher risk of being maltreated than are typical children. The evidence base on the abuse of children with disabilities living in low- and middle-income countries is extremely limited but the problem is particularly acute in East Africa. We don’t know the types of evidence that exist on this topic. This problem is compounded by the fact that key indicators of disability, such as reliable prevalence rates, are not available currently. This paper addresses this serious problem by mapping the existing evidence-base to document the coverage, patterns, and gaps in existing research on the abuse of children with disabilities in East Africa. An evidence map, following systematic review guidelines, was conducted and included a systematic search, transparent and structured data extraction, and critical appraisal. Health and social science databases (Medline, EMBASE, PsychInfo, Taylor&Francis, Web of Science, and SAGE) were systematically searched for relevant studies. A substantive grey literature search was also conducted. All empirical research on the abuse of CWDs in East Africa was eligible for inclusion: Data on abuse was systematically extracted and the research evidence, following critical appraisal, mapped according to the type of abuse and disability condition, highlighting gaps and patterns in the evidence-base. 6005 studies were identified and screened, of which 177 received a full-text assessment. Of these, 41 studies matched the inclusion criteria. By mapping the available data and reports and systematically assessing their trustworthiness and relevance, we highlight significant gaps in the available evidence base. Clear patterns emerge that show a major data gap and lack of research on sexual abuse of children with disabilities and an identifiable lack of methodological quality in many relevant studies. These make the development of a concerted and targeted research effort to tackle the abuse of children with disabilities in East Africa extremely difficult. This needs to be addressed urgently if the abuse of children with disabilities is to be prioritised by the global health community.

## Introduction

Child maltreatment and abuse, which “includes all forms of physical and emotional ill-treatment, sexual abuse, neglect, and exploitation that results in actual or potential harm to the child’s health, development or dignity” [1], continues to be a global burden but not a global health priority. As disability itself is a risk factor for different forms of abuse [2–6], children with disabilities (CWDs) are more likely to be maltreated than nondisabled children [7]. Although progress has been made on evidencing maltreatment of children with disabilities in middle- and high-income countries [8–11], and on violence against children in Africa generally [12–14], much of the latter research has focused on West Africa and Southern Africa [15–17]. Systematic reviews of evidence for the physical, psychological, sexual and systemic abuse of children for low-income countries on the African continent are scarce [18, 19]. Data *specifically on the abuse of CWDs* is almost non-existent for East Africa. This may be because many CWDs in East Africa often receive limited education or no schooling at all and most surveys on child abuse are conducted at university or secondary school level [20–21]. What data has been collected outside of these settings has (in many cases) been left to specific research programmes or projects run by the non-governmental organization (NGO) sector. Consequently, a serious evidence gap exists on the nature of abuse of CWDs in East Africa. To address this pressing need, we need to begin by developing a detailed mapping of the gaps that are present within existing research. This will allow for the informed development of a comprehensive agenda for research and practice.

In this paper, we systematically review, categorise, and map studies on the physical, psychological, sexual and systemic abuse of CWDs in East Africa. We develop a systematic evidence map of the available evidence base that explicitly shows which areas and relationships have received attention and which have not. This work contributes to the field primarily by identifying an evidence gap and presenting the need for it to be taken forward to support the prioritisation of new research in this neglected area.

## Materials and methods

### Research design

Evidence mapping is a rigorous method of research synthesis, which transparently assesses and structures the type of research conducted in relation to a specific research question, to identify and visualize patterns and gaps in the existing evidence base [22–24]. Evidence maps follow accepted guidelines for the conduct of systematic reviews [24], but do not aim to provide a synthesis of the identified evidence-base. Rather, evidence maps present a tool to generate a systematic and transparent overview—most commonly in a visual format—of a body of literature, which has been identified through an exhaustive search and has been subject to a structured coding and quality appraisal process. As such, evidence maps serve as an instrument to support evidence-informed decision-making and guide the prioritization of future research [25]. Depending on the research objective, evidence maps can either be conducted in the process of developing a full systematic review, or likewise operate as a research product in their own right [30]. They have been used to map research evidence that addresses topics including intervention/outcome configurations [25], methodological scope and quality [26] and theories of change [27].

We opted to conduct an evidence map rather than a full systematic review as our research was concerned with providing an overview of the patterns and characteristics of the available evidence base. A systematic review would have allowed us to answer detailed individual questions such as what interventions work to protect children from abuse or why certain types of abuse are more prevalent than other. However, in this evidence map we are more concerned with the higher-level patterns in the evidence-base itself; for example, what research gaps and research clusters exists, and what are their characteristics?

**Fig 1:**
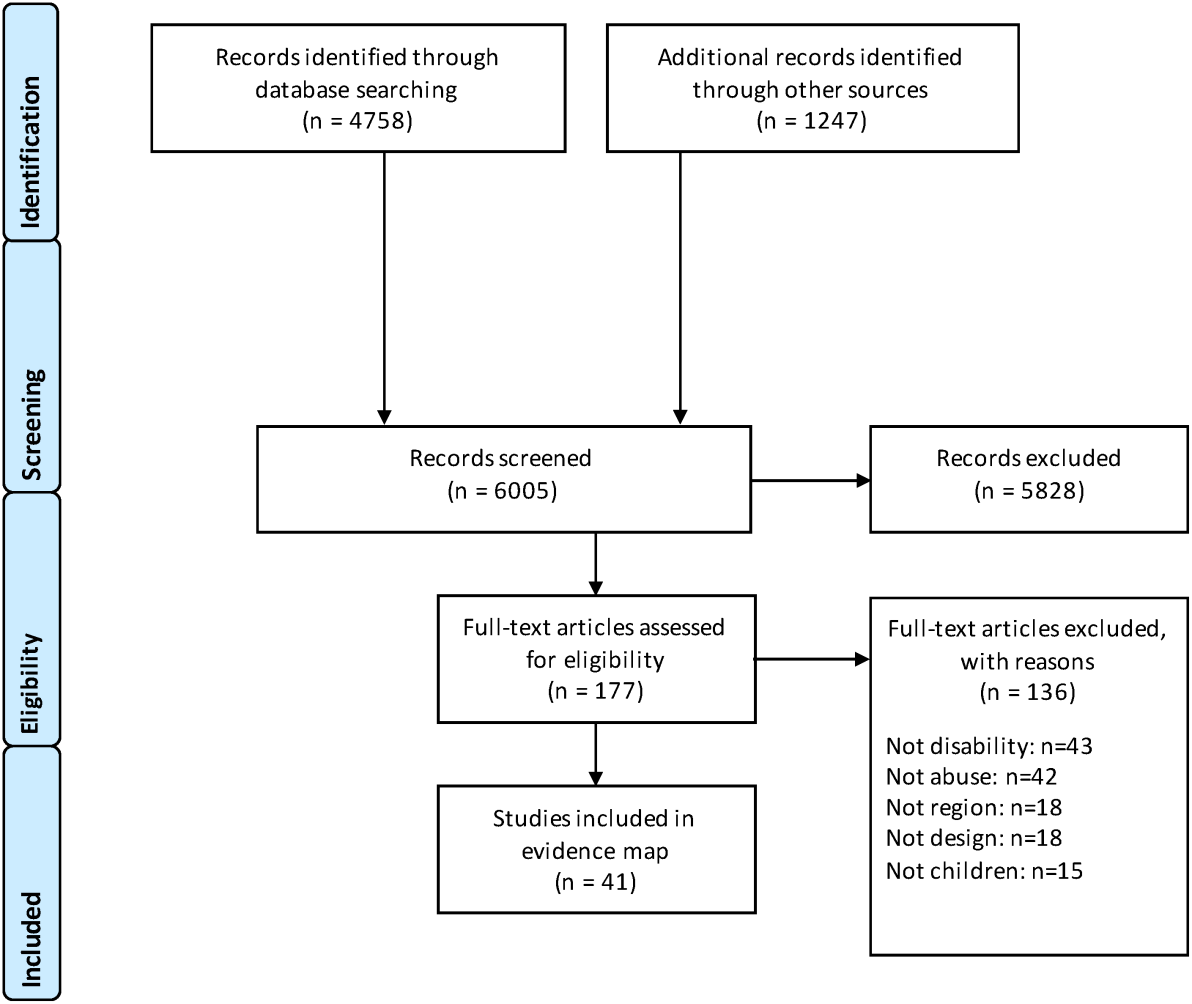
Prisma diagram.

As a result, we conducted a systematic evidence map of all available research investigating the abuse of children with disabilities in East Africa (see Fig 1). We systematically extracted information on different forms of maltreatment and abuse. We then used Heatmap software to map the extracted data and to create a visual overview of *i)* what we know about the relationship between different conditions and the events of abuse; and *ii)* for which conditions and types of abuse there is a lack of research evidence currently. A review protocol was not published and the study was not registered with PROSPERO because these mechanisms do not cater for evidence maps at present.

### Data sources and search strategy for identification of studies

The search strategy to identify relevant studies for the map targeted both academic and grey literature sources to provide an exhaustive account of the evidence base. Academic database searches included health as well as social sciences sources: Medline, EMBASE, PsychInfo, Taylor&Francis, Web of Science, and SAGE. The cut-off date for searches was 1980 and database specific filters (e.g. country filter) were used where applicable. Grey literature sources comprised international and NGO websites (e.g. HandiCap International; UNICEF), government publications, as well as Google and Google Scholar searches. The applied search terms combined terms related to (i) disability; (ii) abuse; and (iii) East Africa using the ‘AND’ boolean operator. Searches were run between August and September 2015. We further applied forward and backward citation searches of all identified studies and screened the reference lists of existing reviews [15,20,21]. Hand-searches of key journals, such as the *East African Medical Journal* were conducted too to ensure the sensitivity of the applied search strategy. An exhaustive record of the full search strategy including key words is provided in S1 Appendix.

### Study selection

A pre-defined set of inclusion criteria was used to identify relevant studies eligible for inclusion in the evidence map. To be included a study had to meet all of the following:

#### Population

Studies eligible for inclusion either had to focus on children with disabilities or the caregivers of children with disabilities. The age definition of “child” adopted by this evidence map follows the UN Convention on the Rights of the Child (CRC) definition [28]. Definitions of disability vary substantially between cultures and societies [29]. To group the studies for the purpose of this evidence map, disabilities were coded according to the WHO’s ICD-10 medical classification. A considerable number of studies did not specify what types of disabilities they were focusing on, and instead referred to ‘disabilities’ more generally. Studies that clustered disabilities together in their statistics were coded in this category.

#### Region

**Fig 2:**
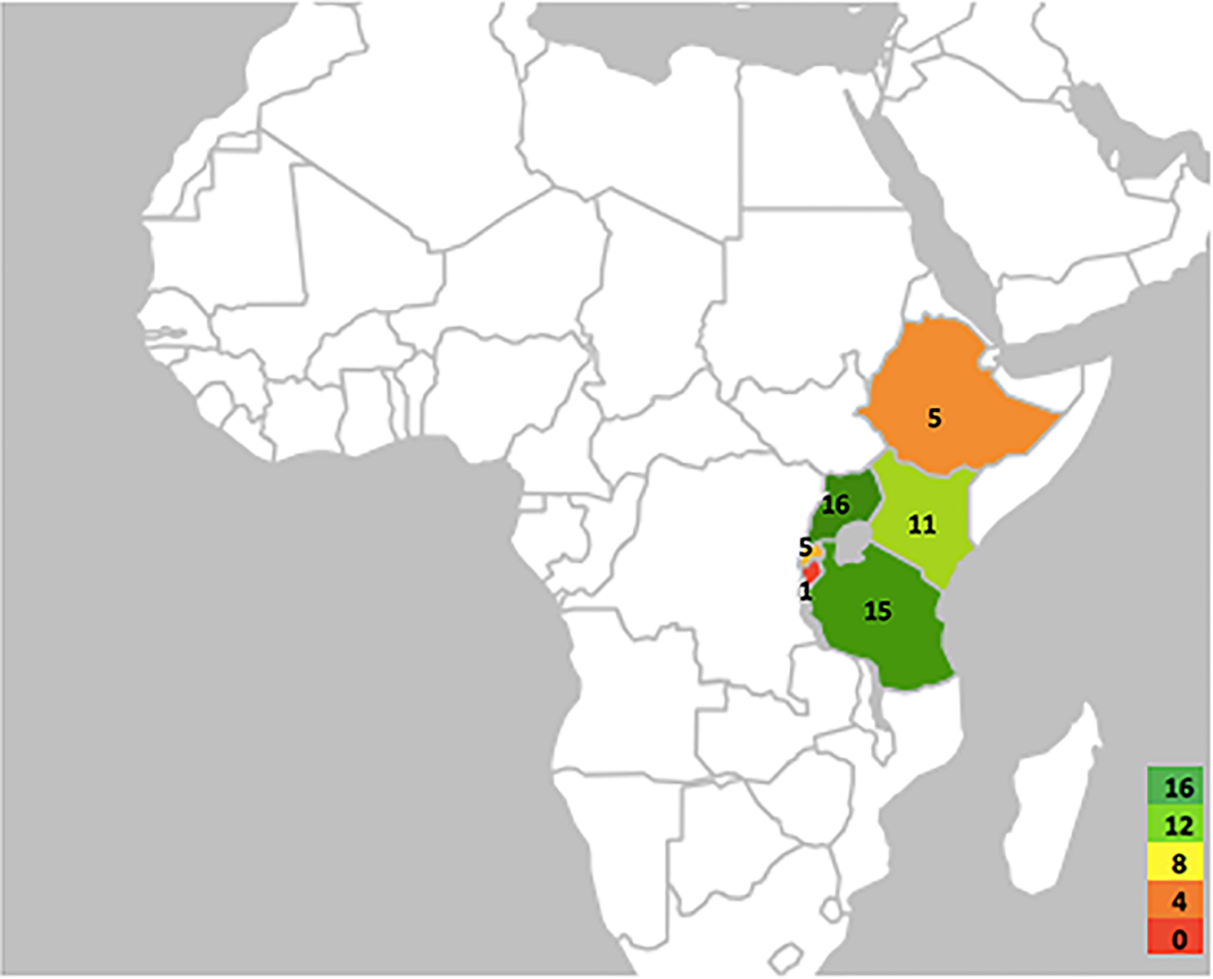
Geographical map of the East African Community indicating the number of studies conducted per country.

Only studies conducted in East Africa were eligible for inclusion in this evidence map. East Africa was defined as the countries belonging to the East African Community: Ethiopia, Kenya, Tanzania, Burundi, Uganda and Rwanda (See Fig 2).

#### Type of abuse

Acknowledging that child maltreatment and abuse can have different definitions across different cultures and societies, the WHO definition of maltreatment (quoted in the introduction) was adopted for this map.

#### Study design

To be included, studies had to present empirical qualitative or quantitative research evidence. Commentary, opinion, advocacy, and theory papers, which were not based on empirical data were excluded. The inclusion threshold of empirical research was met if the research objective, method, population, data, and analysis were described. Eligible study designs can be differentiated into three categories of research: (i) descriptive qualitative and quantitative studies reporting on the abuse/prevention of abuse; (ii) quantitative and qualitative process and impact evaluations of programmes aiming to protect children with disabilities from abuse; (iii) systematic reviews and meta-analyses on (i) or (ii).

### Screening, data extraction, and critical appraisal

#### Screening

Citations identified in the exhaustive search were imported into Endnote and screened on title and abstract for eligibility. Two reviewers conducted the screening and a subset of ten percent of the studies was double-screened to test inter-reviewer reliability. Full-texts of relevant citations were then sought and further assessed against the inclusion criteria following the same test for inter-reviewer reliability. Studies for which the two reviewers could not reach agreement were deferred to a third reviewer, who acted as an arbitrator.

#### Data extraction

Structured data extraction was conducted using a detailed pre-defined coding tool provided in S2 Appendix. Two reviewers applied this tool to systematically investigate the type of abuse reported in each included study. Types of abuse were documented verbatim. Once all data was collected, the reviewers then aggregated the reported abuse from verbatim into descriptive codes, which were further allocated to four overall types of abuse: physical, psychological, sexual, and systemic. This aggregation followed an iterative process but ensured a close link between extracted verbatim codes into descriptive codes to mitigate the risk of researcher-imposed rather than data-driven coding categories. Coding categories were mutually exclusive. Lastly, coded data was transformed into binary codes to allow for software input.

#### Critical appraisal

Included full-texts were then critically appraised regarding their methodological quality and relevance to the research question. For this purpose, a weight of evidence appraisal tool was developed based on a standard tool designed by Gough [30] and applied at a study level. The concept of ‘weight of evidence’ acknowledges that research quality is determined both by the methodological soundness of the research *and* the relevance of the research to the reviews question of enquiry. Assessing the contribution of a study to the review, therefore, depended on its ability to produce unbiased research findings as much as its overlap with the review question and context. This critical appraisal approach is widely used in reviews of social research because of its ability to incorporate contextual factors into the critical appraisal process [31–32]. It attributes a trustworthiness and a relevance score to each study. To assess the trustworthiness of a study’s findings (i.e. methodological quality), criteria related to the study design, data collection, analysis, and reporting were assessed. To assess the relevance of the study’s findings (i.e. relevance to the evidence map’s objective), the nature of abuse, the strength of the link between the abuse and disability, as well as external validity were examined. Each domain—trustworthiness and relevance—was rated on a scale from low to high. No studies were excluded from the evidence map on the basis of the weight of evidence appraisal; that is, all studies even studies rated as low relevance and low trustworthiness, are featured on the evidence map. The critical appraisal was conducted by two researchers (AG and LL) using the same quality assurance processes outlined above regarding the screening of studies for inclusion.

#### Population of evidence map

**Fig 3:**
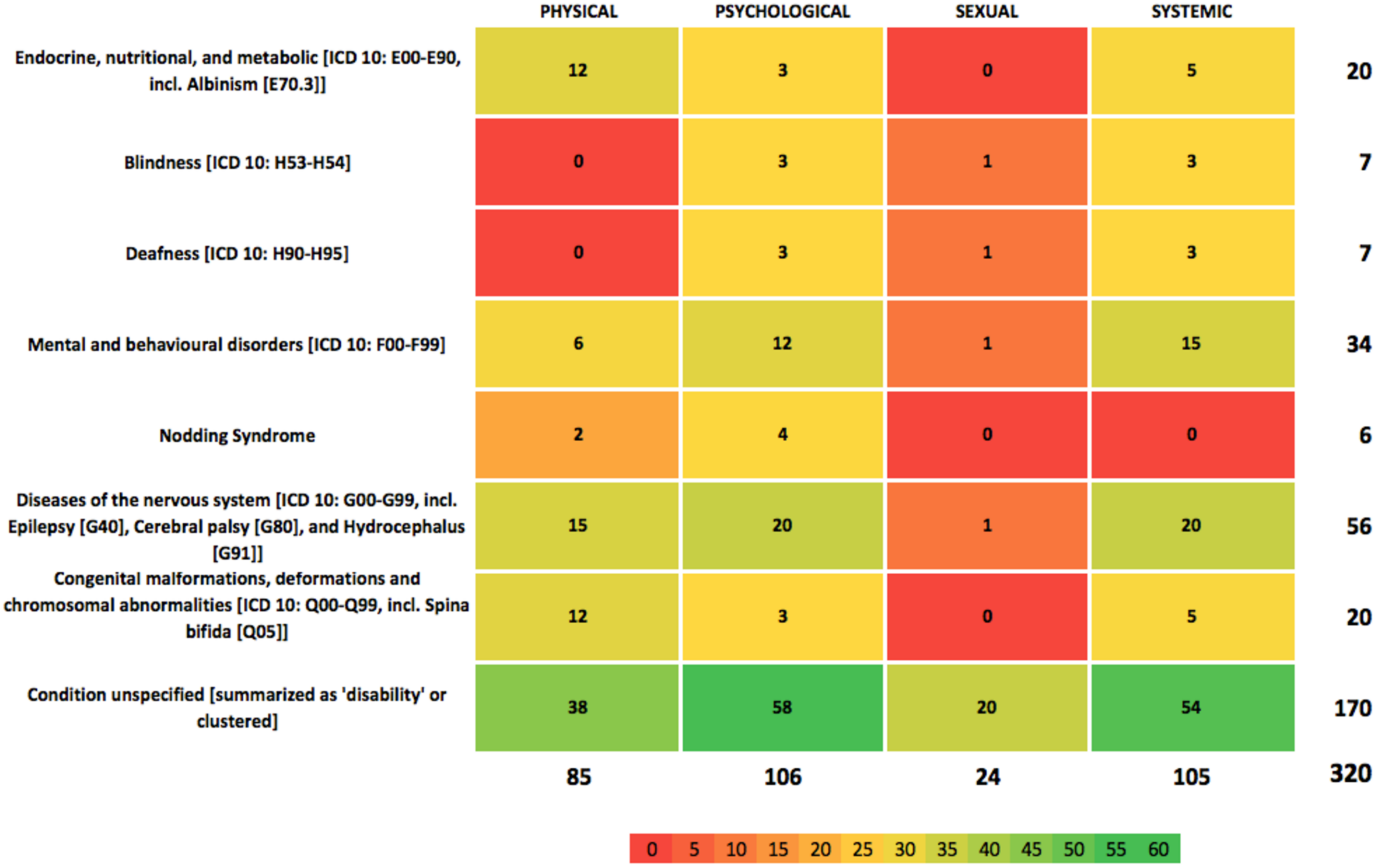
Heatmap of abuse and maltreatment research studies.

**Fig 4:**
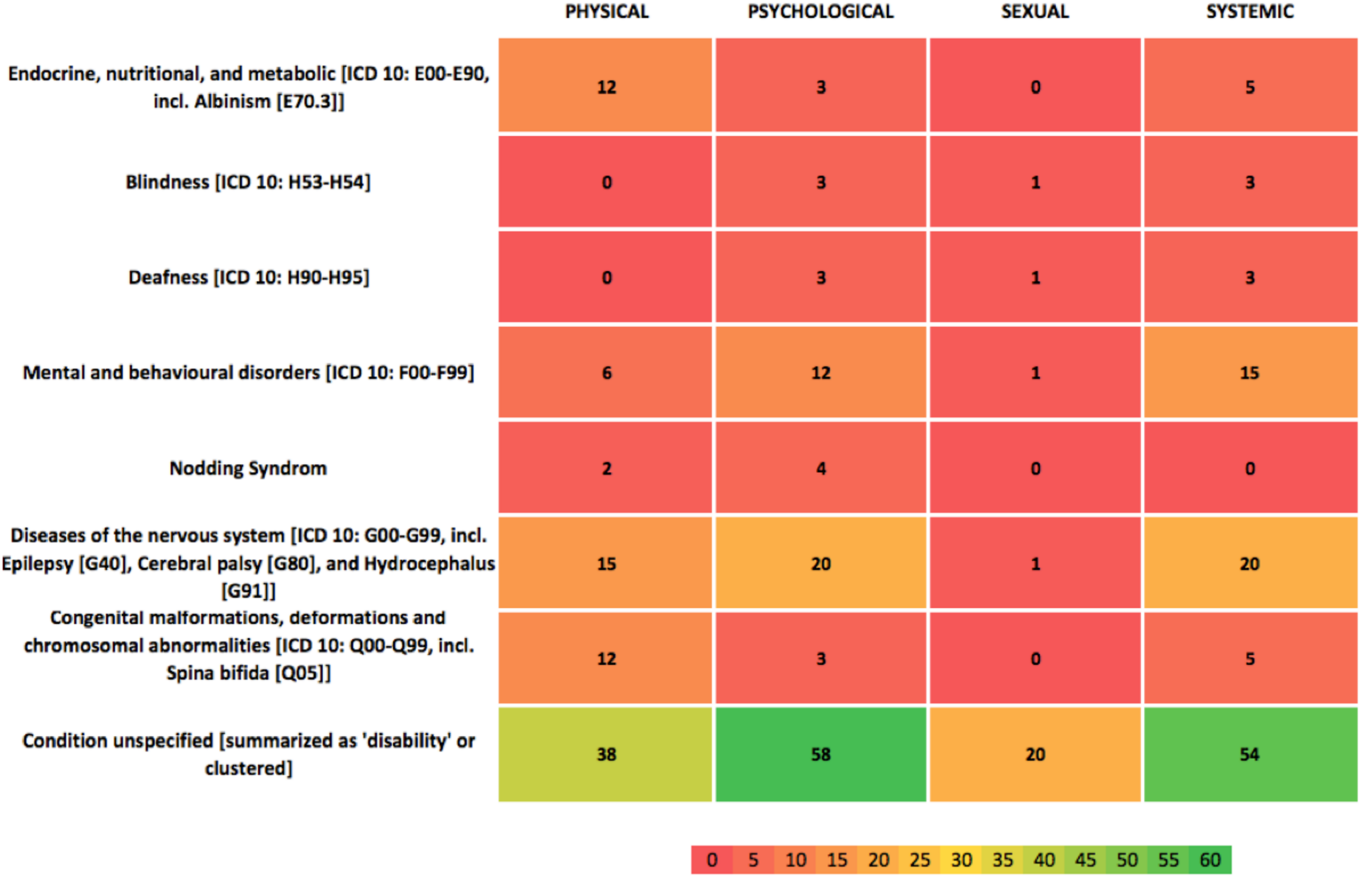
Heatmap with colours indicating percentage of evidence.

Heatmap software in MS Excel was used to generate a visual representation of the identified evidence-base. The binary codes recording instances of abuse served as a data input, with the overall categories of disabilities and abuse presenting the framework for the evidence map. The resulting evidence map (visualized in Figs 3 and 4) hence visually highlights the coverage, patterns, and gaps in the existing research on the abuse of children with disabilities in East Africa. The first heatmap (Fig 3) records the number of instances of abuse or maltreatment for a specific disability. The second heatmap (Fig 4) shows this information as a percentage.

This evidence map is limited in its regional scope and ability to provide a synthesis of effects, such as a meta-analysis. It also included a broader body of evidence than is commonly targeted in systematic reviews of health care research. An interactive and searchable database of the included studies is in production.

## Results

### Search results

The systematic search identified a total of 6,005 citations in the academic and grey literature (see Fig 1). Of these, 5,828 citations were excluded on title and abstract as not relevant, leaving 177 studies eligible to be screened at full-text. Applying the above inclusion criteria, a further 136 studies were excluded. Reasons for exclusions at full-text are presented in Fig 1, with most studies being excluded due to not focusing on children with disabilities (n = 43) and not mentioning instances of maltreatment (n = 42). Additional reasons for exclusion referred to studies not meeting the East Africa inclusion criteria (n = 18), non-empirical studies (n = 18), and studies that talked about maltreatment of disabled people, but not of children under the age of 18 (n = 15). Following this process, we included 41 studies [33–73] in the evidence map. A summary of these 41 studies and their coded key characteristics is provided in S3 Appendix. The PRISMA checklist is provided in S4 Appendix.

### Description of the included studies

Of the total number of the studies that were included (n = 41), 24 were qualitative, five quantitative, eight contained both quantitative and qualitative elements, and four resembled empirical policy analyses. The included studies could be clearly divided into research-based advocacy reports by non-governmental organisations (n = 11) and peer-reviewed academic studies (n = 30). The types of maltreatment and abuse of children with disabilities that emerged from the coding of the studies were clustered into four overarching categories: physical, psychological, sexual and systemic maltreatment and abuse.

### Evidence mapping and identification of key gaps and patterns

Fig 3 categorises 320 instances of research on abuse and maltreatment covered by the 41 included studies. (An interactive version of the heatmap with direct access to information about the included studies and options to tailor the map according to country, type of literature, and weight of evidence assessment can be found at: http://tinyurl.com/ace-evidencemap.) This evidence base is divided into four categories (physical, psychological, sexual and systemic) of abuse and maltreatment for eight disabilities (including where the disability was unspecified or disabilities were aggregated for analysis), presented in a matrix of 32 cells. The number of each cell represents the research evidence reporting instances of abuse or maltreatment for that cell.

The evidence map clearly highlights key gaps, coverage, and structural patterns. Given the differing prevalence rates for each disability, we would not expect each cell to have an equal amount of research evidence. Nevertheless, a number of emerging patterns can be delineated. What is clearly noticeable is that 53% of research treats disability as a *homogenous condition*; in many cases data is not disaggregated by condition or conditions are clustered together when discussed. This is particularly true when discussing blindness and deafness; in some cases they are clustered under disability more generally (bottom column of Fig 3) and in others are discussed under their own categorisation (7 instances each or 2% each of the overall evidence base). This observed aggregation of different conditions as ‘disability’ has clear implications: First, it presents a challenge when using the research to inform practice and policy. Conditions differ in their impact on children with disabilities, the development of their capabilities and their support needs. It would seem a stretch of the research evidence to assume that children with diverse conditions (for example, deafness, epilepsy and spina bifida) can be treated as a homogenous group and that findings regarding the type of abuse resonate across categories. Second, homogeneity does not allow for research findings to inform targeted interventions for children with specific disabilities, suffering particular kinds of abuse and maltreatment. Third, from a practice view, aggregation negates the agency and contexts of children with disabilities who have been abused and can lead to them being treated as “all the same”.

**Fig 5:**
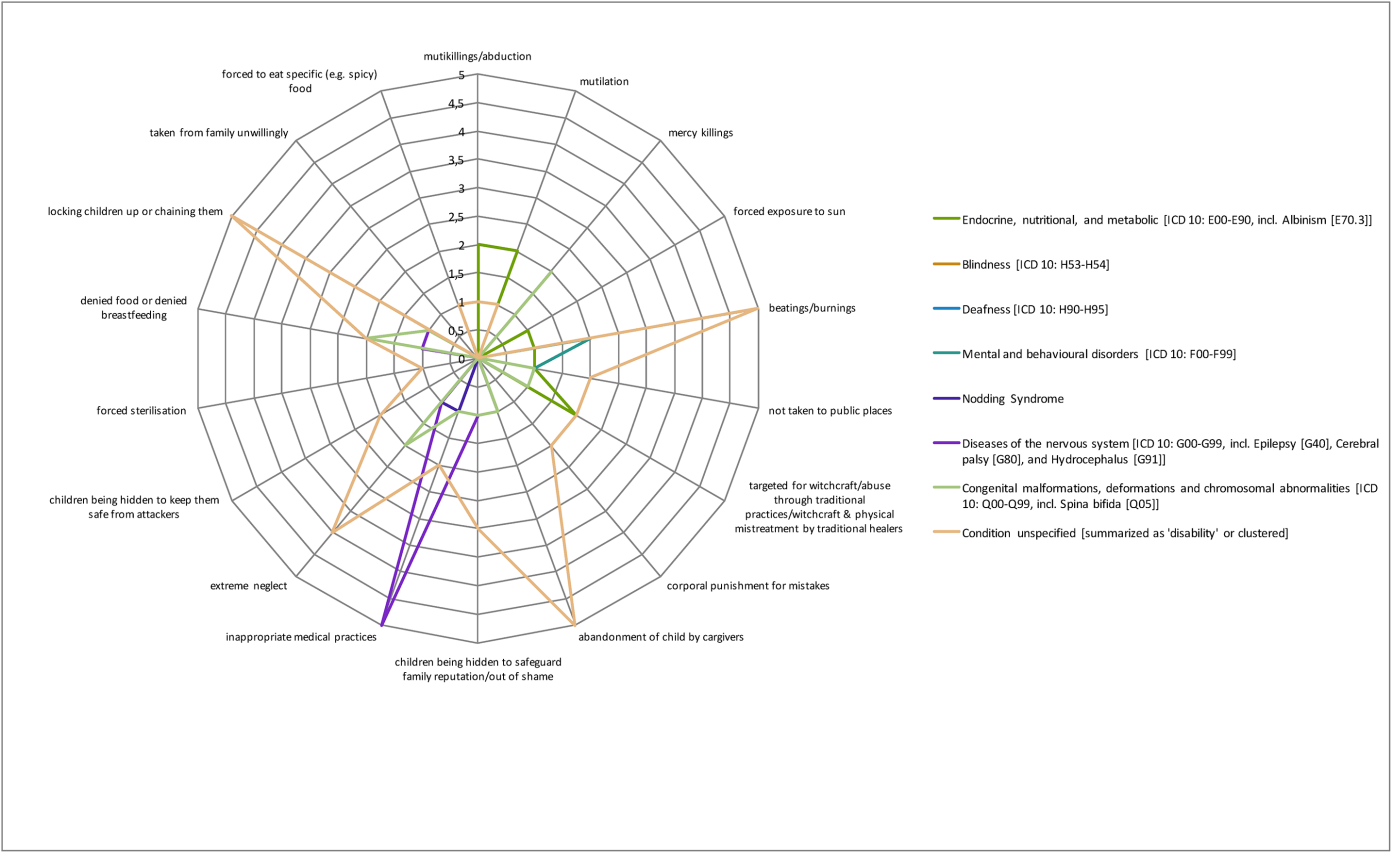
Evidence of physical abuse by type of disability.

**Fig 6:**
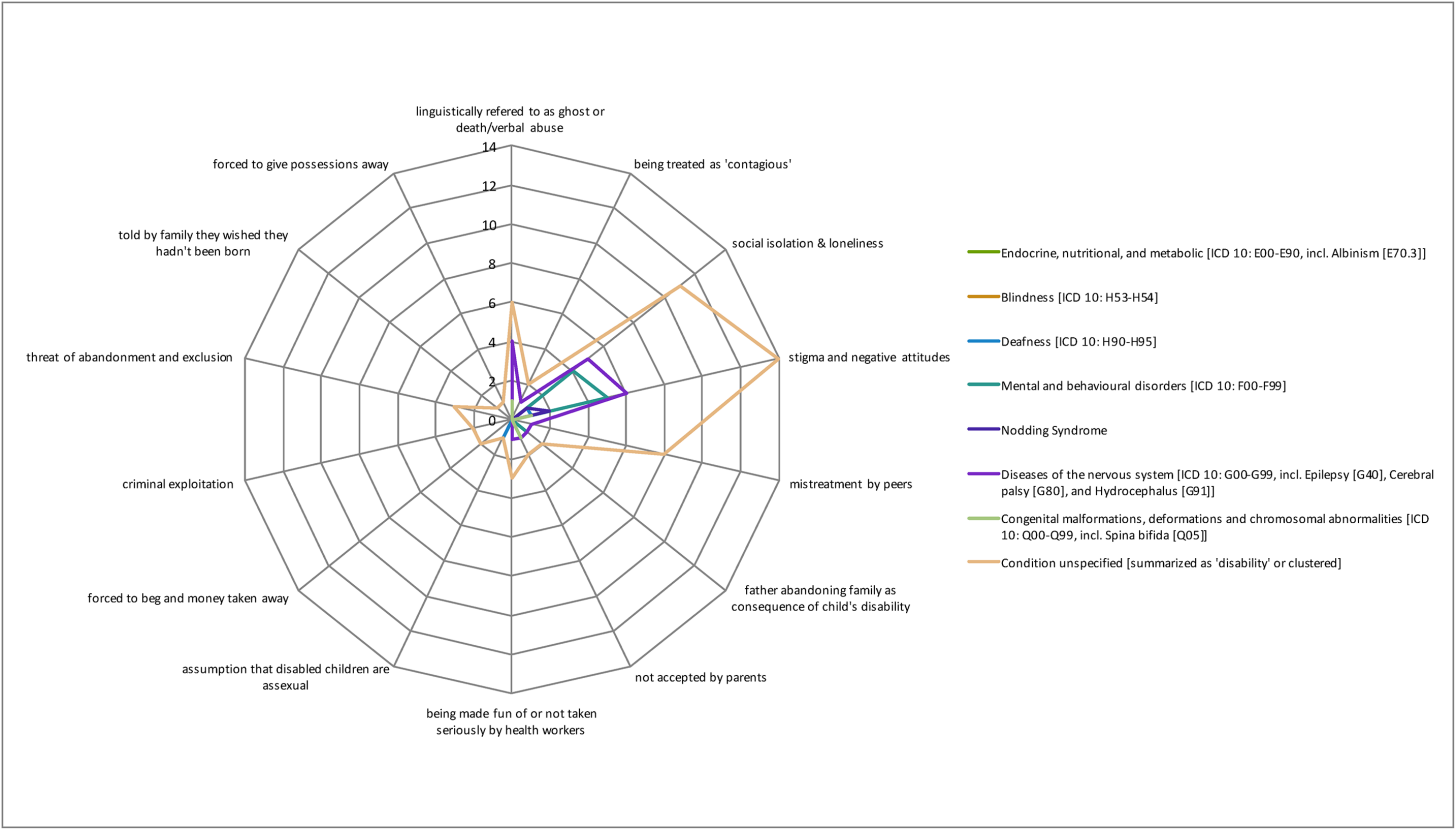
Evidence of psychological abuse by type of disability.

**Fig 7:**
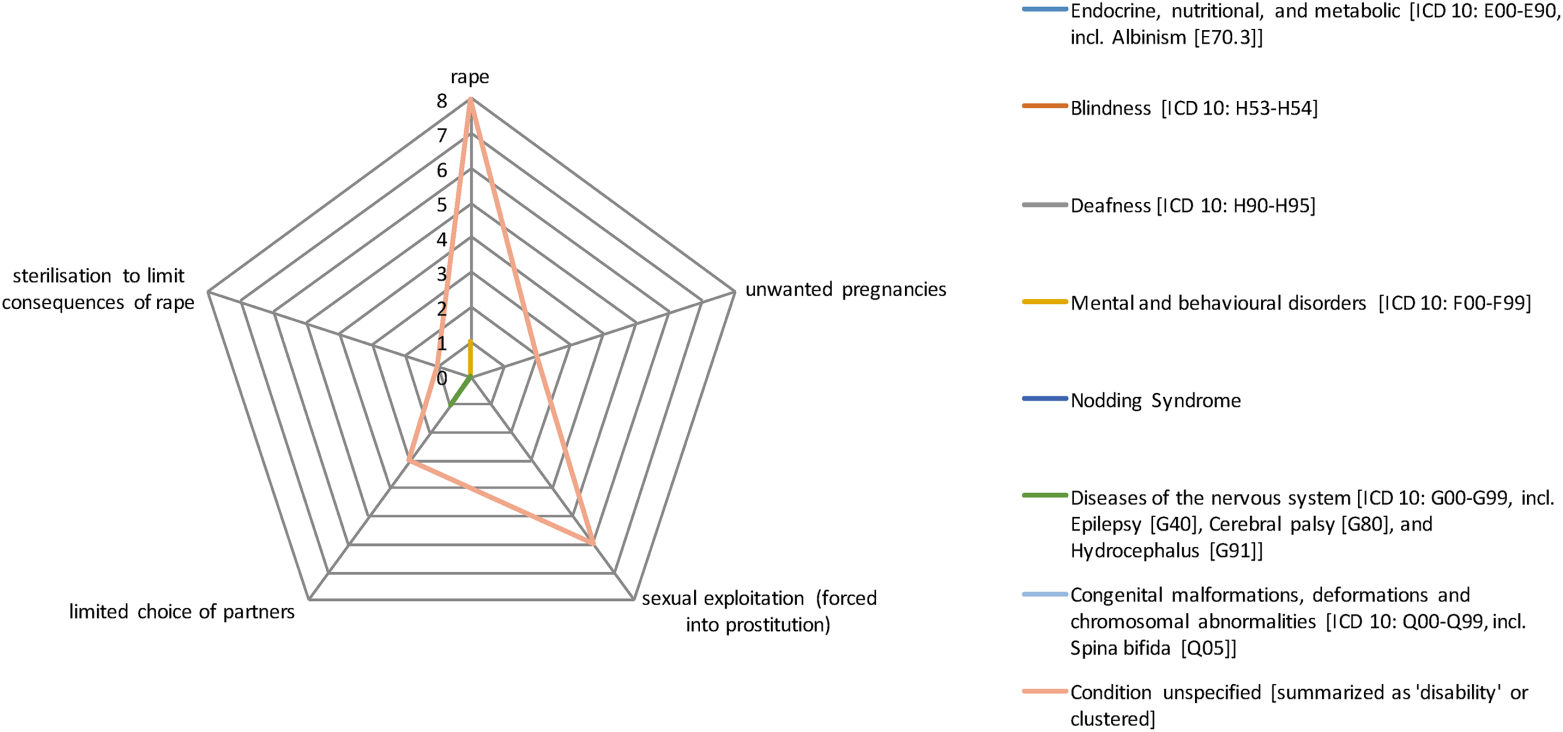
Evidence of sexual abuse by type of disability.

**Fig 8:**
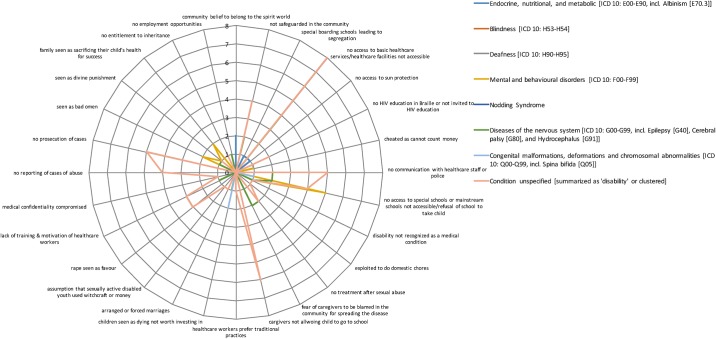
Evidence of systemic abuse by type of disability.

Fig 4 colour-codes the total evidence base in percentage terms, illustrating a real paucity of research (defined as a cell having <5% of the total evidence base (16/320 instances)) across cells. Particularly stark gaps include sexual abuse, which sees the biggest gap in terms of data available (7 out of 8 cells having none or only one study associated with it). Indeed, only 24 instances (7.5%) cover this category as a whole compared to psychological abuse, which has 106 instances (33%) covering mainly unspecified conditions, diseases of the nervous system and mental and behavioural disorders. The radar graphs (Figs 5–8) show a detailed breakdown of the types of abuse and maltreatment for each disability condition in each category.

The radar graph data (see Fig 6) shows that research is primarily driven by studies on stigma and negative attitudes, social isolation and loneliness, and mistreatment by peers. Areas of need are clearly identifiable, including congenital malformation, and endocrine, nutritional and metabolic conditions, both of which have only 3 instances (<1%) associated with them. There is relatively good coverage of systemic abuse (105 instances or 33%) and physical abuse (85 instances or 27%). A wide range of systemic acts of maltreatment or abuse are reported (see Fig 8) including limited access to healthcare and school, cases of rape not being reported or prosecuted and lack of health worker training. Physical abuse too takes many forms including killing, mutilation, extreme neglect, burnings and abandonment (see Fig 5 for specific details). Coverage in this category is particularly strong for diseases of the nervous system, congenital malformations and endocrine, nutritional and metabolic conditions (see Fig 3).

Overall coverage with respect to conditions (see Fig 3) is middling and certainly far from ideal. For example, diseases of the nervous system (56/320 or 17%) and mental and behavioural disorders (34/320 or 11%) are relatively well covered. However, it is clear that some relationships have not been explored, in particular with respect to sexual abuse. There is also a gap on the correlation between mental health issues and physical and sexual abuse. Furthermore, a clear gap exists on abuses that generate disabilities; we did not find any studies or statistics on how (multiple) impairments may develop as a result of maltreatment or abuse.

### Tensions between trustworthiness and relevance in the evidence base

**Fig 9:**
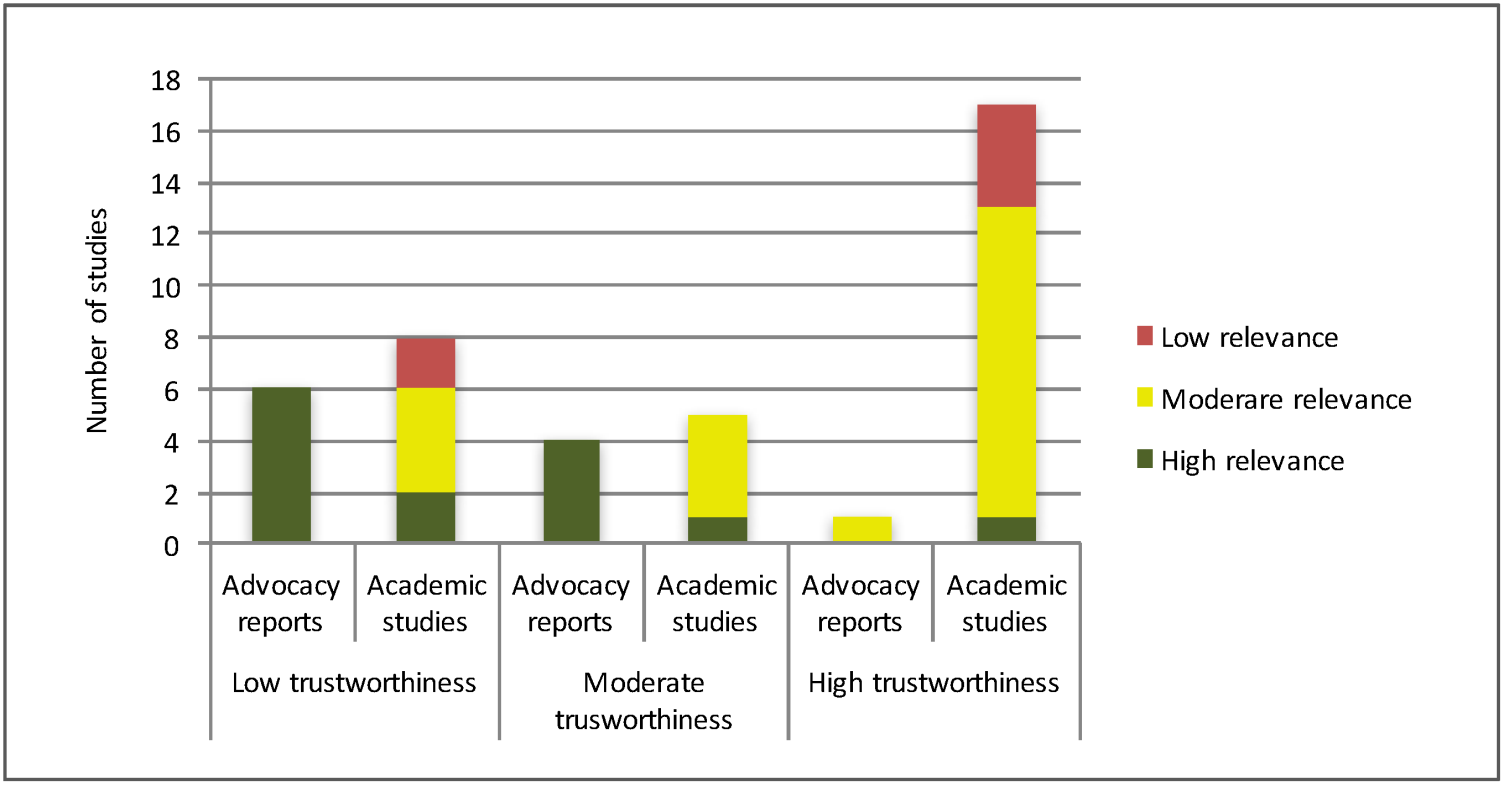
Weight of the evidence.

As detailed in the methods section, included full-texts were critically appraised regarding their methodological quality and relevance to the research question. Fig 9 presents the results of this critical appraisal. Only a single study [33] was rated as high for both trustworthiness and relevance. In other words, the evidence map barely identified any research evidence that applies a rigorous methodological design to investigate the abuse of disabled children in East Africa. Analysing the critical appraisal in more detail, a clear pattern emerges: studies of high relevance tended to be of lower trustworthiness and vice versa. This pattern was correlated with the particular type of research conducted. Research-based advocacy reports (i.e. research conducted by advocacy groups and NGOs such as HandiCap International with the explicit purpose to motivate policy change) were more often of high relevance but lower trustworthiness (i.e. methodological quality), while peer-reviewed studies were more often of high trustworthiness but of lower relevance to the issue of maltreatment and abuse. Overall, given the studies found through our exhaustive research, most of which only tangentially report on maltreatment and abuse and mention the issue only in passing, there appears to be little data on the issue of abuse and maltreatment of children with disabilities in East Africa.

As shown in Fig 9, of the 14 studies identified as highly relevant, ten were research-based advocacy reports, while of the 18 studies that fell into the category of ‘high trustworthiness’, 17 were peer-reviewed academic studies. More than half of the advocacy reports (n = 6) contained findings of high relevance, but low trustworthiness. The clear implication of this is that highly relevant research on abuse and maltreatment of children with disabilities in East Africa is being left to advocacy groups to undertake. Academics clearly need to work more closely with practitioners to ensure improved methodological quality, while acknowledging major gaps in their own research agendas.

## Discussion

Within global health “there are few mechanisms to capture what practitioners learn in the field and thereby add to a shared store of knowledge about effective care delivery in settings of privation” [74]. Through the development of the evidence map, this paper has explored the characteristics of and key gaps in the evidence base regarding the abuse and maltreatment of children with disabilities in East Africa. Based on these findings, we suggest that this can help guide the prioritisation of future research in this area in the following areas:

1. *The challenge of programme design*: It is clear from our analysis that significant key gaps remain in our understanding of the abuse and maltreatment of children with disabilities in East Africa. Given this, a major challenge remains in designing effective programmes and interventions when little or no data exists on needs and contexts of abused and maltreated children with specific disabilities.
2. *Positioning abuse of children with disabilities as a priority for global health*: Shiffman and Smith [75] developed a framework with four categories of factors that influence whether a global health issue attracts political priority: (i) the power of actors involved, (ii) ideas used to portray the issue, (iii) the nature of the political contexts in which the actors operate, and (iv) characteristics of the issue itself. Regarding factors (i) and (ii) specifically, the high levels of stigmatisation of disability adversely effects public portrayals of the issue, leading to a lack of resonance with communities. The differing nature of the research base recorded for academic and practitioner communities indicate a lack of linkages between these actors, and the impact on their collective capacity to fight for the rights of children with disabilities who have been abused or maltreated. With respect to factor (iv), Shiffman and Smith [75] point out that credible indicators (i.e. “clear measures that show the severity of the problem and that can be used to monitor progress”, p. 1371) need to be available for an issue to attract political priority. Our evidence map shows clearly where they are not and thus point to remaining work to be done. They also point to the need for effective interventions, the lack of which we discussed above. Finally, our evidence map highlights the real lack of knowledge with respect to the severity of the abuse burden relative to other health issues, thereby rendering claims around the issue more difficult to make.
3. *Develop a visionary research agenda*: More empirical research and more robust evidence is needed with regard to prevalence rates, reporting mechanisms, and awareness raising among doctors and healthcare workers more generally. As our study has shown, at present, research is largely driven by advocacy organisations, which present evidence that is of less rigorous methodological quality. This undermines the pressing nature of this issue. As a result, it does not gain sufficient attention in the global health community. For researchers, there is a need to undertake this work despite its consensus challenging and uncomfortable nature, and an increased need for Africa-led international collaborations among researchers.

## Conclusion

Making evidence-informed claims on the abuse of children with disabilities in East Africa remains a challenge. More empirical research and more robust evidence are needed, specifically with regard to prevalence rates, reporting mechanisms, and awareness raising amongst healthcare workers. Currently, research is largely left to and driven by advocacy organisations, resulting in evidence that is of less rigorous methodological quality than academic research. This undermines the pressing nature of this issue within the global health community. International institutions need to prioritise research on strengthening the evidence base on the abuse of children with a wide variety of disabilities. This can then form the basis for the development of targeted interventions to safeguard these children. Our evidence gap map is a productive step in achieving this aim.

## Supporting information

S1 Appendix The exhaustive record of the full search strategy including key words. (DOCX)

S2 Appendix The pre-defined coding tool. (XLSX)

S3 Appendix A summary of the 41 studies and their coded key characteristics. (XLSX)

S4 Appendix The PRISMA checklist. (DOC)
