# Supplementary material for: Physical, psychological, sexual, and systemic abuse of children with disabilities in East Africa: Mapping the evidence
Source: PLoS One. 2017 Sep 11;12(9):e0184541. doi: 10.1371/journal.pone.0184541 (PMC5593191; doi:10.1371/journal.pone.0184541)
Supplement: S1 Appendix — (DOCX) [file pone.0184541.s001.docx]

**Taylor & Francis**

| 32 | Disability AND Abuse AND Africa |
| --- | --- |
| 3169 (in everything) [0 in abstract] | Disability AND Abuse AND (East Africa OR Eastern Africa OR Ethiopia OR Tanzania OR Rwanda OR Uganda OR Kenya OR Burundi)  SAME results for  Disability AND (Abuse OR physical abuse OR emotional abuse OR sexual abuse OR psychological abuse) AND (East Africa OR Eastern Africa OR Ethopia Tanzania OR Rwanda OR Uganda OR Kenya OR Burundi) |
| 4051 (in everything) | (Disability OR handicap OR abnormal OR paralysed) AND (Abuse OR physical abuse OR emotional abuse OR sexual abuse OR psychological abuse OR exploitation OR witchcraft OR infanticide OR abduction OR punishment OR aggression OR maltreatment OR neglect OR rape OR forced sex OR incest OR stigma OR community violence) AND (East Africa OR Eastern Africa OR Ethiopia OR Tanzania OR Rwanda OR Uganda OR Kenya OR Burundi) |
| 1237 | "spina bifida" OR neurodisability OR neurodisabilities OR epilepsy OR "cerebral palsy" OR hydrocephalus OR rickets OR "neurodegenerative disorders" OR Albino OR Albinism  **AND**  ("East* Africa" OR Ethiopia OR Kenya OR Tanzania OR Uganda OR Burundi OR Rwanda) |

**Web of science**

| 13 | ((Disability OR disabilities OR disabled) AND Abuse AND (East* Africa OR Ethiopia OR Kenya OR Tanzania OR Uganda OR Burundi OR Rwanda)) |
| --- | --- |
| 1624 | (Abuse OR physical abuse OR emotional abuse OR sex* abuse OR psychological abuse OR exploitation OR witchcraft OR magic OR traditional OR healer OR victim* OR infanticide OR community OR abduction OR kill* OR corporal* OR punishment OR aggression OR violence OR maltreatment OR neglect OR rape* OR forced sex OR incest OR fondling* OR stigma* OR community violence) **AND** (handicap* OR delay* OR abnorm* OR neuro* OR paralyze* OR disab* OR retard*OR spirit OR snake OR mental OR learning OR difficult*) **AND** (East* Africa OR Ethiopia OR Kenya OR Tanzania OR Uganda OR Burundi OR Rwanda) **AND** (child* OR adolescent* OR boy* OR girl* OR baby OR youth OR young OR teen OR puberty OR pre-puberty OR minor OR juvenile OR toddler OR infant OR newborn*) |
| 697 | (Abuse OR “physical abuse” OR “emotional abuse" OR "sex* abuse" OR "psychological abuse" OR exploitation OR witchcraft OR magic OR "traditional healer" OR victim* OR infanticide OR abduction OR kill* OR corporal* OR punishment OR aggression OR violence OR maltreatment OR neglect OR rape* OR "forced sex" OR incest OR fondling* OR stigma* OR "community violence") **AND** (handicap* OR delay* OR abnorm* OR paralyze* OR disab* OR retard* OR spirit OR snake OR mental OR "learning difficult*") **AND** ("East* Africa" OR Ethiopia OR Kenya OR Tanzania OR Uganda OR Burundi OR Rwanda) |
| 606 | "spina bifida" OR neurodisability OR neurodisabilities OR epilepsy OR "cerebral palsy" OR hydrocephalus OR rickets OR "neurodegenerative disorders" OR Albino OR Albinism  **AND**  ("East* Africa" OR Ethiopia OR Kenya OR Tanzania OR Uganda OR Burundi OR Rwanda) |

**Ebsco**

| 14 | ((Disability OR disabilities OR disabled) AND Abuse AND (East* Africa OR Ethiopia OR Kenya OR Tanzania OR Uganda OR Burundi OR Rwanda)) |
| --- | --- |
| 425 | ((Disability OR disabilities OR disabled) AND Abuse AND (**Africa** OR Ethiopia OR Kenya OR Tanzania OR Uganda OR Burundi OR Rwanda)) |
| 54 | (Disability OR disabled OR handicap OR abnormal OR paralysed) AND (abuse OR exploitation OR witchcraft OR infanticide OR abduction OR punishment OR aggression OR maltreatment OR neglect OR rape OR forced sex OR incest OR stigma OR community violence) AND (East* Africa OR Ethiopia OR Kenya OR Tanzania OR Uganda OR Burundi OR Rwanda) |
| 411 | "spina bifida" OR neurodisability OR neurodisabilities OR epilepsy OR "cerebral palsy" OR hydrocephalus OR rickets OR "neurodegenerative disorders" OR Albino OR Albinism  **AND**  ("East* Africa" OR Ethiopia OR Kenya OR Tanzania OR Uganda OR Burundi OR Rwanda) |

**SAGE**

| 1 | Disability or disabled in Abstract and abuse in Abstract and ("east Africa" "eastern Africa") or (Ethiopia OR Tanzania OR Uganda OR Kenya OR Burundi OR Rwanda) in all fields, |
| --- | --- |
| 213 | abuse and disab* in all fields and East Africa OR ethiopia OR tanzania OR uganda OR kenya OR rwanda in all fields, |
| 67 | "spina bifida" OR neurodisability OR neurodisabilities OR epilepsy OR "cerebral palsy" OR hydrocephalus OR rickets OR "neurodegenerative disorders" OR Albino OR Albinism  **AND**  ("East* Africa" OR Ethiopia OR Kenya OR Tanzania OR Uganda OR Burundi OR Rwanda) |

**Medline & PsychInfo**

| 29 (age filter for 0-18 for Medline) search in abstract  No difference if filter is removed | ("East* Africa" OR Ethiopia OR Tanzania OR Rwanda OR Uganda OR Kenya OR Burundi)  AND  (handicapped OR delayed OR abnormal OR disability OR disabled OR paralyze* OR retarded)  AND  (spirit OR snake OR exploitation OR witchcraft OR infanticide OR abduction OR kill OR aggression OR violence OR abuse OR maltreatment OR neglect OR rape OR "forced sex" OR incest OR stigma OR "community violence") |
| --- | --- |
| 116 search in all text | ("East* Africa" OR Ethiopia OR Tanzania OR Rwanda OR Uganda OR Kenya OR Burundi)  AND  (handicapped OR delayed OR abnormal OR disability OR disabled OR paralyze* OR retarded)  AND  (spirit OR snake OR exploitation OR witchcraft OR infanticide OR abduction OR kill OR aggression OR violence OR abuse OR maltreatment OR neglect OR rape OR "forced sex" OR incest OR stigma OR "community violence") |

**Citation Searches: 747**

**Google & Google Scholar: first 500 each**

**Hand-searched:**

- Disability, CBR & Inclusive Development (hand search)
- East African Medical Journal (searched on disability AND abuse terms)
- African Journal of Disability (hand search)

Website searches:

- WHO
- UNICEF
- African Child Policy Forum
- Handicap International
